# Electronic Properties of Fluoride and Half–fluoride Superlattices KZnF_3_/KAgF_3_ and SrTiO_3_/KAgF_3_

**DOI:** 10.1038/srep15849

**Published:** 2015-10-30

**Authors:** Xiaoping Yang, Haibin Su

**Affiliations:** 1 Institute of Advanced Studies, Nanyang Technological University, 50 Nanyang Avenue, 639798 Singapore.

## Abstract

We present the formation of cupratelike electronic structures in KAgF_3_–related superlattices resulted from the confinement together with structural chemical modification by using the generalized gradient approximation augmented with maximally localized Wannier functions analysis. Strong antiferromagnetic coupling found in bulk KAgF_3_ is held in purely–fluoride KZnF_3_/KAgF_3_. Under 4% in–plane compression strain, its Fermi surface shape breaks away from the edge of electron pocket and resembles that of La_2_CuO_4_. While within half–fluoride SrTiO_3_/KAgF_3_, out–of–plane electronic reconstruction results in electron doping of AgF_2_ plane and antiferromagnetic state instability, and the Fermi surface shape presents considerable similarity to that in HgBaCuO_4_. These results shed light on two dimensional antiferromagnetic precursors of a new Ag^II^ family of high–temperature superconductors.

Motivated by recent susceptibility measurements of D. Kurzydlowski *et al.*^1^, which indicates that KAgF_3_ compound exhibits strong antiferromagnetic (AFM) coupling reminiscent of that found in copper(II) oxides^2^,^3^, we carried out investigation on electronic properties of fluoride and half–fluoride KAgF_3_–related superlattices (SLs). For a long time, the discovery of high–temperature superconductivity in under doped cuprates^4^ initiated the quest for finding related transition–metal compounds with possible superconductivity. Ag^2+^ is isoelectronic to Cu^2+^ (*d*^9^ configuration). F^−^ and O^2−^ are also isoelectronic ions, closed–shell species. Moreover, both F^−^ and O^2−^ are weak–field ligands. Previous theoretical studies^5^,^6^,^7^ of Grochala and Hoffmann have also suggested that properly hole– or electron–doped Ag^II^ fluorides might be good superconductors, due to similarity in structure and properties between the Ag^II^ fluorides and the cuprate superconductors.

[CuO_2_]∞ plane with tetragonal tetra coordination of Cu (weak apical Cu–O bonds), is an essential structural element for superconductivity in cuprates. Analogous [AgF_2_]∞ plane with Ag centers in a tetragonal tetra coordination is still not known experimentally. However, benefit from recent development of heterostructure interface technology, superlattices containing Ag^II^F_2_ square lattices can be prepared by using appropriate synthetic techniques. Superlattice is consisted of alternating layers of different transition metal compounds^8^,^9^,^10^,^11^,^12^,^13^,^14^,^15^, even technically a single atomic layer can be inserted at interface^12^. Here, interface can be used to modulate electronic structure for manipulating physical properties and generating novel phases which are not present in the bulk constituents^16^,^17^,^18^,^19^,^20^. In our paper, our research focus on artificial superlattice materials design and their electronic properties, different from research on real bulk Ag^II^ fluorides materials^5^,^6^,^7^.

We investigate electronic structures, magnetic states, model hamiltonian parameters and effective Fermi surfaces (FSs) for purely–fluoride and half–fluoride superlattices KZnF_3_/KAgF_3_ and SrTiO_3_/KAgF_3_, as illustrated in the top panels of Fig. 1, and compare these with corresponding properties of the cuprate superconductors. These fluorides exhibit cupratelike band structures and strong AFM fluctuations. The energy bands around the Fermi level are sensitive to in–plane strain, and the FS shapes present considerable similarity to those in cuprates. Model hamiltonian parameters are extracted and compared to La_2_CuO_4_ (LCO), HgBa_2_CuO_4_ (HBCO). Strong AFM coupling found in bulk KAgF_3_ is held in purely–fluoride KZnF_3_/KAgF_3_. While half–fluoride SrTiO_3_/KAgF_3_ is at the edge of superconducting transition, in which FM state becomes much high in energy, and AFM state is just below nonmagnetic (NM) state by only 11.675 meV/Ag due to out–of–plane electronic reconstruction. Our finding suggests that fluoride and half–fluoride KAgF_3_–related SLs indeed have the potential to become 2D AFM precursors of a new Ag^II^ family of high-temperature superconductors.

## Results

For the in–plane lattice constant *a*, we took that of KZnF3 (4.068 Å) for purely–fluoride KZnF_3_/KAgF_3_ SL, and took that of STO (3.905 Å) for half–fluoride SrTiO_3_/KAgF_3_ SL. The lattice constant *c* and atomic *z* coordinates were fully relaxed. The structural difference between two kinds of SLs results from different polarization strength in neighboring atomic layers of AgF_2_ plane. Negatively charged F and positively charged K cation are displaced relative to each other in KF atomic layers, and thereby polarize the cation and anion planes so as to affect apical Ag–F bond length. AgF_2_ layer acts as the mirror plane of whole unit cell.

A large cation–anion polarization occurs in KF plane of half–fluoride SrTiO_3_/KAgF_3_, and fluorin atoms move symmetrically against AgF_2_ plane by 0.163 Å. This polarization distortion produces a local ionic dipole moment, and together with in–plane strain it leads to a large apical Ag–F distance 
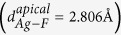
. This apical Ag–F bond length is more close to those of cuprates than recent reported 3.405 Å for SrTiO_3_/CsAgF_3_ SL^20^, due to the smaller size of the K^+^ cation. However, in purely–fluoride KZnF_3_/KAgF_3_ SL, polarization distortion is weak, and is just 0.004 Å toward AgF_2_ plane. As a result, apical Ag–F bond length is smaller than that in SrTiO_3_/KAgF_3_ by 0.298 Å.

**Figure 1:**
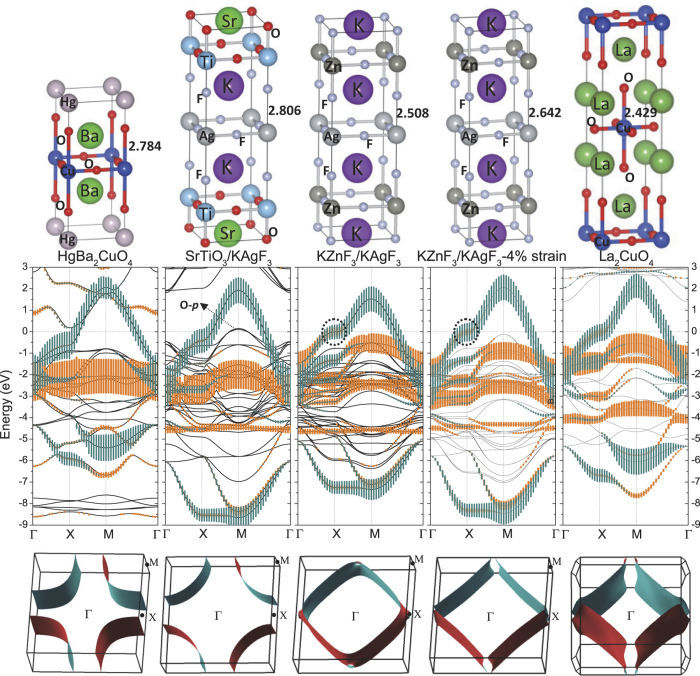
Schematic geometrical structures, GGA bandstructures and the effective the Fermi surfaces centered at Γ point in first Brillouin Zone from 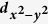 band for bulk HBCO, SrTiO_3_/KAgF_3_, KZnF_3_/KAgF_3_ without and with in–plane compression strain, bulk LCO from left to right. The Fermi level *ε*_*F*_ is set at zero. Dark cyan and orange fatbands represent contribution of 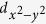 and 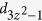 orbitals respectively.

An evolution of Ag-*e*_*g*_ states with structural chemical modification can be clearly observed in band structures of Fig. 1. Local ionic dipole moment perturbs electrostatic potential and changes band positions around the Fermi level. Spin–polarized GGA calculations give nonmagnetic ground state for both superlattices. Figure 1 shows energy bands of SrTiO_3_/KAgF_3_ and KZnF_3_/KAgF_3_ SLs in a 12 eV region around the Fermi level *ε*_*F*_ ≡ 0 and along the symmetry–lines 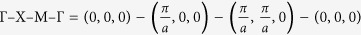. The energy bands of bulk LCO and HBCO are also plotted in Fig. 1 for comparison. For SLs, electronic properties around *ε*_*F*_ are still mainly controlled by Ag-*e*_*g*_ bands, which are above the filled O/F-2*p* and Ag-*t*_2*g*_ bands, and below the empty Ti-3*d*/Zn-4*s* bands. We plot 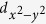 (dark cyan) and 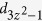 (orange) fatbands around *ε*_*F*_ to disclose their orbital contribution. For KZnF_3_/KAgF_3_ SL, 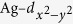 antibonding band is just below the Fermi level at X point, and resembles that of LCO. But Ag–*e*_*g*_ antibonding band’s width is smaller than that of LCO and HBCO. Since electronic properties are subject to electron– and orbital–lattice couplings in perovskite–like materials, similar calculation is made for KZnF_3_/KAgF_3_ SL with an additional in–plane lattice constants of 3.905 Å. Energy bands are found to be sensitive to in–plane strain, and this 4% compression strain increases band width close to that of LCO. However, in SrTiO_3_/KAgF_3_ case, the antibonding band between 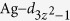 and F-*p* states disappears due to the weak mixing of Ag-3*d* and F-*p* states in *z* direction. *e*_*g*_ bands from −3 to 2 eV appear more like that of HBCO with a larger apical Cu-O distance of 2.784 Å. Most importantly, atomic polarization results in oxygen 2*p* band edge of TiO_2_ plane upshift eventually above the Fermi level and charge transfer with 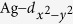 band, as occurs in SrTiO_3_/CsAgF_3_ SL^20^. The FSs centered at Γ point for LCO, HBCO and KAgF_3_-related SLs are shown in the third row of Fig. 1. Compared to LCO (transition temperature *T*_*c*_ = 40 K), the FS of HBCO (*T*_*c*_ = 90 K) has the typical shape of high-*T*_*c*_ cuprates superconductor with constant–energy surface obviously bulging toward Γ point. The FS shape of KZnF_3_/KAgF_3_ without strain is at the edge of electron pocket and far away from that of HBCO or LCO. But the FS under 4% compression strain looks more like that of LCO. However, for STO/KAgF_3_ with polarized electron–doping in AgF_2_ plane, effective FS from 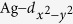 band presents the considerable similarity to that of HBCO.

Next, we discuss the stability of magnetic states in superlattices under GGA + *U*_*d*_ scheme. AFM band structures indicate that KZnF_3_/KAgF_3_ SL presents a AFM insulating ground state with a energy gap of 0.445 eV. A 4% compression strain decreases energy gap to 0.232 eV. For SrTiO_3_/KAgF_3_, an AFM metallic ground state is obtained, which is aroused by charge transfer between O-*p*_*x*_, *p*_*y*_ orbitals in TiO_2_ plane and covalent hybrid orbitals of 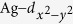 and F-*p*_*x*_, *p*_*y*_ in AgF_2_ plane. In Table 1, we summarize in–plane and apical bond lengths 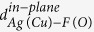 and 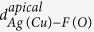, energy difference *E*_*FM*_ − *E*_*AFM*_, and magnetic moment on Ag/Cu atom in AFM state. The calculated nearest neighboring magnetic exchange coupling constant J (~(*E*_*FM*_ − *E*_*AFM*_)/Cu) for LCO and HBCO is in qualitative agreement with the value derived from two–magnon scattering experiments [J_*expt*_ = 128 meV]^21^. Generally, strong AFM coupling is held in heterostructure configuration with a confined 2D [AgF_2_]∞ plane. The obtained J value (~(*E*_*FM*_ − *E*_*AFM*_)/Ag) in undoping purely–fluoride KZnF_3_/KAgF_3_ SLs is close to that found in bulk KAgF_3_ (~100 meV)^1^, but smaller than related cuprates (see Table 1) due to less localized in 4*d*-orbitals of Ag. And in–plane compression strain increases *E*_*FM*_ − *E*_*AFM*_ from 90.305 meV/Ag to 101.605 meV/Ag, similar to the trend for cuprates (*e.g.* from 127.8025 meV/Cu for HBCO to 177.465 meV/Cu for LCO in Table 1). Our finding suggests that fluoride KAgF_3_ related SLs indeed have the potential to become precursors of a new family of high-temperature superconductors which could benefit from enhancement of the critical superconducting temperature due to strong magnetic fluctuations^22^. In half–fluoride SrTiO_3_/KAgF_3_ SL, FM state becomes much high in energy and unavailable. AFM state is just below NM state by only 11.675 meV/Ag due to out–of–plane electronic reconstruction.

Based on the aboved GGA simulations, we extract model hamiltonian parameters by MLWFs downfolding technique. Fourier transformation of the orthonormalized MLWE Hamiltonian *H*(*k*), yields on–site energies and hopping integrals


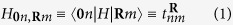


in a Wannier representation, where 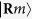 is orthonormal MLWF Wannier function in cell **R** associated with band *m*, and 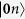 is MLWF Wannier function in home cell associated with band *n*.

**Figure 2:**
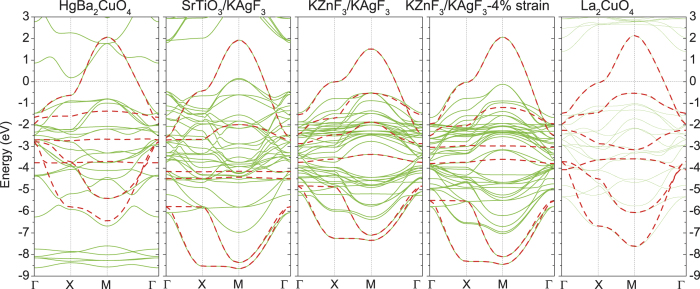
Effective *e*_*g*_ MLWF bands (red dash lines) for bulk HBCO, SrTiO_3_/KAgF_3_, KZnF_3_/KAgF_3_ without and with in–plane compression strain, bulk LCO superimposed to the GGA electronic bands (green solid lines). The Fermi level *ε*_*F*_ is set at zero.

We choose to downfold to a 6-band hamiltonian describing the in-plane 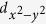, *p*_*x*_, *p*_*y*_ orbitals, and out–of–plane 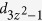, two *p*_*z*_ orbitals. In particular, four parameters capture the essential physics: the *e*_*g*_ crystal field splitting energy 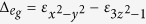, the in–plane charge–transfer energy 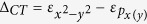, the direct in–plane Ag–F hopping *t*_*pd*_, and the shortest–ranged in–plane F–F hoppings *t*_*pp*_. The extracted values are tabulated in Table 2, and corresponding interpolated band structure are shown in Fig. 2.

The hopping integrals *t*_*pd*_ and *t*_*pp*_ of LCO and HBCO are in good agreement with the 3–band model results^23^,^24^ and the analysis of the photoelectron spectroscopy^25^. While the data of Δ_*CT*_ are further corrected in our model by including three additional out–of–plane orbitals. Compared to cuprates, purely–fluoride KZnF_3_/KAgF_3_ has relatively larger 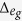, Δ_*CT*_, and in–plane Ag–F hopping *t*_*pd*_, and smaller hopping *t*_*pp*_. In–plane compression strain increase the values of the former three parameters 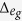, Δ_*CT*_ and *t*_*pd*_, but has only a slight change on the hopping *t*_*pp*_. Under the same in–plane lattice constant 3.905 Å, half–fluoride SrTiO_3_/KAgF_3_ has obvious larger 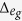, Δ_*CT*_ and slightly increased *t*_*pp*_, compared to purely–fluoride KZnF_3_/KAgF_3_. Across cuprate families, the charge transfer energy is an increasing linear function of Madelung potential difference for a hole between the copper and in–plane oxygen, and correlate with the maximum superconducting transition temperature *T*_*c*,*max*_^26^. The decreasing Δ_*CT*_ correlates with a enhanced *T*_*c*,*max*_. Here, half–fluoride SrTiO_3_/KAgF_3_ has a slight reduced Δ_*CT*_ value 3.459 eV between the silver and in–plane fluorine, compared to the reported 3.504 eV for SrTiO_3_/CsAgF_3_ 20, while purely–fluoride KZnF_3_/KAgF_3_ has a obvious smaller charge transfer gap, as shown in Table 2.

## Discussion

In conclusion, we investigate cupratelike electronic structures and strong AFM fluctuations effect in the proposed KAgF_3_–related superlattices. Compared to bulk KAgF_3_, undoping purely–fluoride KZnF_3_/KAgF_3_ SL has a similar magnetic coupling constant. A 4% in–plane compression strain stabilizes AFM state further, and makes the FS shape to deviate from the edge of electron pocket and to resemble that of LCO. In half–fluoride SrTiO_3_/KAgF_3_ SL, atomic polarization induces out–of–plane electronic reconstruction occurring between O-*p*_*x*_, *p*_*y*_ orbitals in TiO_2_ plane and covalent hybrid orbitals of 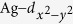 and F-*p*_*x*_, *p*_*y*_ in AgF_2_ plane, which results in AFM state instability by a smaller energy difference *E*_*NM*_ − *E*_*AFM*_ of 11.675 meV/Ag. And FS shape of half–fluoride SL presents considerable similarity to that in HBCO. Therefore, fluoride and half–fluoride KAgF_3_–related superlattices indeed have the potential to become 2D AFM precursors of a new Ag^II^ family of high–temperature superconductors, which could benefit from enhancement of the critical superconducting temperature due to strong magnetic fluctuation, and the relative small charge transfer gap in KZnF_3_/KAgF_3_.

## Method

We carried out the numerical calculations using the Vienna *ab initio* Simulation Package (VASP)^27^,^28^,^29^,^30^ within the framework of the generalized gradient approximation (GGA) (Perdew-Burke-Ernzerhof exchange correlation functional)^31^, and recently developed maximally localized Wannier functions (MLWFs) downfolding technique^32^,^33^,^34^. The ion–electron interaction was modeled by the projector augmented wave (PAW) method^35^,^36^ with a uniform energy cutoff of 500 eV. Spacing between *k* points was 0.02 Å^−1^. The geometrcal structures of the SLs were optimized by employing the conjugate gradient technique, and in the final geometry, no force on the atoms exceeded 0.01 eV/Å. For magnetic states calculations, we used *U*_*d*_ = 7.5 eV and *J*_*d*_ = 0.98 eV for Cu-3*d* state^37^ and a smaller *U*_*d*_ = 5 eV and *J*_*d*_ = 0.98 for Ag-4*d* state^38^.

## Additional Information

**How to cite this article**: Yang, X. and Su, H. Electronic Properties of Fluoride and Half-fluoride Superlattices KZnF_3_/KAgF_3_ and SrTiO_3_/KAgF_3_. *Sci. Rep.* **5**, 15849; doi: 10.1038/srep15849 (2015).

## Figures and Tables

**Table 1:** The in–plane and apical bond length 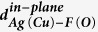 and 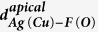 in Å, energy differences *E*_*FM*_ − *E*_*AFM*_ in meV/Ag(Cu), and Ag/Cu atom's magnetic moment of AFM state in *μ*_*B*_/Ag(Cu), for LCO, HBCO, KZnF_3_/KAgF_3_ without and with strain, SrTiO_3_/KAgF_3_.

	LCO	HBCO	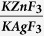	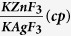	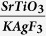
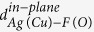	1.894	1.941	2.034	1.953	1.953
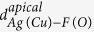	2.429	2.784	2.508	2.642	2.806
*E*_*FM*_ − *E*_*AFM*_	177.465	127.8025	90.305	101.605	11.675*
Moment	0.542	0.495	0.442	0.447	0.268

Here, *cp* between parentheses is the abbreviation for “compression”.

^*^Substituted by *E*_*NM*_ − *E*_*AFM*_ since FM state becomes unavailable.

**Table 2:** Tight–binding parameters of the six–band *p*-*d* model, containing the in–plane 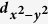 , *p*_*x*_, *p*_*y*_ orbitals and out–of–plane 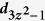 , *p*_*z*_ orbitals for LCO, HBCO, KZnF_3_/KAgF_3_ without and with in–plane strain, SrTiO_3_/KAgF_3_.

	LCO	HBCO	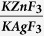	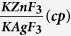	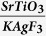
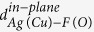	1.894	1.941	2.034	1.953	1.953
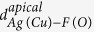	2.429	2.784	2.508	2.642	2.806
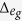	0.005	0.115	0.095	0.227	0.477
Δ_*CT*_	2.305	1.476	2.624	3.247	3.459
*t*_*pd*_	1.395	1.249	1.483	1.754	1.756
*t*_*pp*_	0.656	0.620	0.350	0.400	0.415

Parameters include *e*_*g*_ crystal field splitting energies 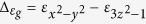, charge–transfer energies 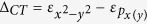, the two nearest–neighbor (intra–cell) hoppings *t*_*pd*_, *t*_*pp*_ in eV. The in–plane and apical bond length 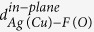 and 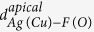 in Å are also listed to identify structural chemical difference. Here, *cp* inside parentheses is the abbreviation for “compression”.
